# Heat‐induced structural changes in fish muscle collagen related to texture development in fish balls: Using eel ball as a study model

**DOI:** 10.1002/fsn3.2462

**Published:** 2021-07-19

**Authors:** Yafei Wang, Xufeng Wang, Chuntong Lin, Mengqin Yu, Shanshan Chen, Jingke Guo, Pingfan Rao, Song Miao, Shutao Liu

**Affiliations:** ^1^ College of Biological Science and Engineering Fuzhou University Fuzhou China; ^2^ Joint Centre for Food and Nutrition Research SIBS, CAS‐Zhejiang Gongshang University Hangzhou China; ^3^ Teagasc Food Research Centre Moorepark Cork Ireland

**Keywords:** collagen, fish ball, heating, structure, texture

## Abstract

In order to elucidate the substantial effect and underlying mechanism of endogenous collagen on the texture development of fish balls, the structural and gelling properties of eel muscle collagen (EMC) under different heat treatments, as well as their effects on texture of eel ball, were investigated. EMC resulted in significant improvement of eel ball texture via gelling ability, filler effect, and interaction with starch. Under mild heating below 90°C for 30 min, the structural and physicochemical changes of EMC varied gradually, resulting in improved storage modulus of starch‐containing myofibrillar gel, a mimic of eel ball. However, overheating (100°C, 30 min) induced EMC degradation and significantly decreased the gel formation and the improvements in textural properties. Supplementation of EMC to eel balls significantly improved its gel strength, springiness, cohesiveness, and chewiness, as well as uniformity and tightness of the microstructure. These results suggest the texture development of eel ball can be regulated by heat‐induced structural changes, as well as structure–function relationship of collagen, compared with previous studies on myofibrillar proteins and exogenous gelatin; and they may provide texture‐related insights to the quality control of fish balls and diverse heat‐treated products of surimi containing collagen.

## Graphical abstract

1. Heat‐induced structure changes of eel muscle collagen (EMC) in eel ball were verified and were closely related to the texture of eel ball. 2. Mild heating of EMC improved the textural profile of mimic eel ball; overheating induced EMC degradation and decreased the gel forming ability. 3. Overheating‐induced EMC degradation decreased textural improvement in eel ball.

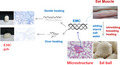

## 1 INTRODUCTION

Collagen, a major component of connective tissue in fishes, is sensitive to heat treatment. Denaturation of fish collagen occurs between 15 and 45°C, which probably involves the breakage of hydrogen bonds, resulting in loss of fibrillar structure followed by contraction of the collagen molecule (Veeruraj et al., 2013). In fish species, collagen is also one of the important determinants of texture, Sato et al. (1986) found a strong correlation between the collagen content and toughness of raw meat in different fish species. During heat processing, the structural and physicochemical changes of collagen have a substantial effect on the texture of certain aquatic products. For example, the tenderness of heated abalone meat is superior to raw abalone meat due to heat‐induced degradation of collagen (Gao et al., 2001; Zhu et al., 2011). Mizuta, Yamada & Miyagi (2010) reported that the histological changes in collagen were related to textural development of prawn meat during heat processing. Additionally, the addition of exogenous fish gelatin, the heat‐degraded collagen (Asai et al., 2019), improved gel strength of Alaska pollock surimi (Hernandez‐Briones et al., 2009), or the texture by decreasing the hardness and chewiness, without affecting the myofibrillar nanostructure of fish balls (Feng, Fu et al., 2017).

Fish ball, a type of spherical gel of fish surimi mixed with starch and salt, is popular in East and Southeast Asia, as well as in Nordic countries. The improvement of the textural properties such as gel strength of fish balls along with the underlying mechanism of action has been a cause for concern with the increasing consumption in recent years (Carmen Gómez‐Guillén et al., 1996; Feng, Fu et al., 2017; Seymour et al., 1997). Fish balls processing usually involves mixing of the materials, such as minced surimi, starch, and salt, and rolling the mixture into a ball, followed by boiling in hot water and cooling. Generally, myofibrillar protein is the dominant factor for the formation of fish ball contributing to the gel strength, whereas as a major component of the extracellular matrix in fish muscle, the effect of collagen on the quality of fish ball has not yet been fully understood. The previous studies (Asai et al., 2019; Feng, Fu et al., 2017; Seymour et al., 1997; Veeruraj et al., 2013) suggest that collagen of fish muscle undergoes heat‐induced structural changes during the boiling, which may affect the texture of fish balls. However, whether the potential heat‐induced structural changes in the intrinsic collagen of fish muscle substantially affect the texture of fish balls and the underlying mechanism of action remains to be elucidated.

In the present study, eel ball, a typical representative of fish balls derived from marine eel muscle, was used as a study model. It has been reported that marine eel muscle contains high collagen content (Saito et al., 2014; Sato et al., 1986; Veeruraj et al., 2013). The acid‐soluble collagen and pepsin‐soluble collagen in marine eel showed *T_d_* of 39 and 35°C, respectively (Veeruraj et al., 2013). To investigate the structural changes of intrinsic collagen during heating and their effects on fish ball texture, the EMC was isolated from marine eel, its heat‐induced structural changes and the effects on physicochemical properties, including protein surface hydrophobicity, hot‐water solubility, average particle diameter, and rheological behavior of EMC gel, were determined. Meanwhile, changes in the texture of eel balls supplemented with EMC and collagenase were investigated. Furthermore, the effect of supplementation of EMC heated in different temperature on the dynamic rheological properties of starch‐containing myofibrillar gel, a mimic of eel ball, was also measured.

The results might be of great importance in elucidating the texture development of eel ball based on heat‐induced structural changes and functionality of endogenous collagen, compared with previous reports highlighting myofibrillar aspects and exogenous gelatin (Feng, Zhu, et al., 2017; Zhang et al., 2013), and provide texture‐related insights to manage the processing and quality control of fish balls and diverse heat‐treated products of fish surimi containing collagen or gelatin. Because the heat‐induced gelation mechanism of fish muscle proteins cannot be totally understood without knowing how combinations of myofibrillar and collagen affect gel properties (Sun & Holley, 2011).

## 2 MATERIALS AND METHODS

### 2.1 Materials

Eel (*Muraenesox cinereus* Forsskal, 1775), salt, and potato starch were purchased from a local market in Fuzhou, south east China. The eels were alive when they were bought from the market, and the ordinary muscle was excised from the eels immediately after decapitating, bloodletting, and removal of internal organs by the market and then stored at −20°C until use. 1,8‐anilino naphthalene sulfonate (ANS) and the standard hydroxyproline were purchased from Millipore Sigma. Acetic acid, potassium bromide (KBr), Masson staining fluid, and dimethylbenzene were purchased from Sangon Co. All other chemicals used were of analytical grade.

### 2.2 Preparation of eel muscle collagen (EMC)

The preparation of EMC was based on the method of Veeruraj et al. (2013), with slight modifications. Muscle tissue (about 1,000 g) was homogenized with 3 volumes (v/w) of 0.1 mol/L NaOH in a Hamilton mixer. The suspension was stirred overnight and centrifuged (RX 15, Hitachi Limited) at 10,000 g for 20 min at 4°C, followed by the addition of 10 times the volume of 0.1 mol/L NaOH to the residue and stirring of the suspension overnight. This procedure was repeated three times. The residue after alkali extraction was washed with cold water and extracted with 0.5 mol/L acetic acid at 4°C for 48 hr with stirring. The soluble collagen solution was isolated by centrifugation at 10,000 g for 1 hr. EMC was isolated via salt precipitation with 0.9 mol/L NaCl in 0.5 mol/L acetic acid. The purified collagen was dissolved in 0.5 mol/L acetic acid, dialyzed against 0.1 mol/L acetic acid, and lyophilized.

### 2.3 Differential scanning calorimetry (DSC) of EMC

DSC of EMC was performed according to the method described by Kittiphattanabawon et al. (2005) with slight modification. The EMC was rehydrated by adding 0.5 mol/L acetic acid to dried samples at a solid/solution ratio of 1:10 (w/v). DSC was performed using a differential scanning calorimeter (DSC3, METTLER TOLEDO). The sample (4.0 mg) was accurately weighed into an aluminum pan and sealed and then scanned at 10°C/min in the range of 20–65°C under liquid nitrogen as the cooling medium. The empty pan was used as the control. The peak maximum value is the maximum transition temperature (T_max_), and the peak area is the total denaturation enthalpy (DH) according to the DSC thermogram.

### 2.4 Circular dichroism (CD) measurement

The lyophilized EMC was dissolved in 0.5 mol/L acetic acid and stirred at 4°C overnight, followed by centrifugation at 500 g for 5 min at 4°C. The solution was finally diluted to the protein concentration of 0.1 mg/ml with 0.5 mol/L acetic acid. The solutions were heated at 25, 30, 40, 60, 90, and 100°C for 30 min. The sample was placed in a quartz cell with a path length of 0.1 cm and scanned at 190–280 nm at a speed of 20 nm/min using the circular dichroic instrument (J‐1500, JASCO). Each sample was scanned three times. The average molar ellipticity (θ) was calculated according to the molar mass of the residue, and the unit was expressed as deg × cm^2^/dmol.

### 2.5 Fourier transform infrared spectroscopy (FTIR)

The EMC was analyzed using FTIR (Nicolet 380; Thermo Fisher Scientific). The lyophilized EMC (3 mg) was mixed with dried KBr (100 mg), ground in an agate mortar, and pressed into a tablet. The disk was placed in the temperature change accessory of the FTIR instrument. The scanning temperature points were 25, 40, 60, and 90°C, with incubation for 30 min at each temperature. The scan range was 400–4,000 cm^−1^, and the resolution was 4 cm^−1^. Using the baseline method to calculate the absorption intensity of the peaks, the resultant spectra were analyzed using the Origin 2017 software (Origin Lab).

### 2.6 Determination of surface hydrophobicity

The protein surface hydrophobicity of EMC was determined according to the method of Wang et al. (2017) using ANS as the probe, with slight modifications. The EMC was dissolved in 10 mmol/L, pH 7.0 phosphate buffer with concentrations of 0.2, 0.4, 0.6, 0.8, and 1.0 mg/ml and heated at 25, 40, 60, and 90°C for 30 min. A 10 µl aliquot of ANS stock solution (8 mmol/L) was added to 2.0 ml of the heated sample solution. The fluorescence intensity of ANS conjugates was measured using a spectrofluorometer (FluoroMax‐4; HORIBA) at an excitation wavelength of 390 nm and an emission wavelength of 470 nm. The relative fluorescence intensities (FI) of each sample were then acquired by subtracting the FI attributed to collagen in the buffer. The initial slope of the FI versus collagen concentration (mg/ml) was calculated via linear regression analysis and reported as an index of surface hydrophobicity of EMC.

### 2.7 Measurement of hot‐water solubility

The solubility of collagen in water was measured over the temperature range 30–100°C as described by Mizuta et al. (1995). EMC was suspended in 20 mmol/L sodium phosphate buffer, pH 7.2, at a concentration of 0.1% (w/v) and homogenized at 500 rad/min for 2 min using a microhomogenizer. The homogenate was heated at 30, 40, 50, 60, 70, 80, 90, or 100°C for 30 min in a water bath and centrifuged at 10,000 g for 20 min. The hydroxyproline content in the supernatants and the unheated EMC was determined using the method of Woessner (1961). The hot‐water solubility of the EMC was expressed as the proportion of hydroxyproline in the soluble fraction relative to the total hydroxyproline.

### 2.8 Measurement of average particle diameter

Average particle diameter was determined via dynamic light scattering (Zetasizer Nano ZS 90; Malvern Instruments). The EMC samples were diluted to 3 mg/ml with 20 mmol/L sodium phosphate buffer, pH 7.2, heated at 50, 60, 70, 80, 90, or 100°C for 30 min in a water bath and filtered through a 0.45 µm Millipore membrane. The specimens were measured in triplicate.

### 2.9 Electrophoresis

Sodium dodecyl sulfate–polyacrylamide gel electrophoresis (SDS‐PAGE) was carried out according to Wang et al. (2017). 2 mg/ml EMC samples was mixed with 0.125 mol/L, pH 8.0 Tris‐HCl buffer, which contains 10% (v/v) glycerol, 2% (w/v) SDS, 0.05% (w/v) bromophenol blue, and 10% β‐mercaptoethanol (β‐ME). The stacking gel and separating gel were 5% and 12%, respectively.

### 2.10 Rheological measurements

In order to investigate the effect of temperature on gelling of EMC, oscillatory rheological experiments were performed using the rheometer (MCR 302; Anton Paar) via circular plate geometry (d = 50 mm), with the gap set at 1 mm. The dynamic temperature sweep was conducted within the linear range at a constant strain of 1% (according to our preliminary experiment by amplitude sweep) and a frequency of 1 Hz. The EMC samples were diluted to 3.0% (w/v) with distilled water. The temperature sweep was programmed as follows: heating from 25 to 40, 60, 90, or 100°C, respectively, at the rate of 5°C/min, maintaining these temperatures for 10 min, followed by cooling from 40, 60, 90, or 100°C to 4°C at the rate of 5°C/min and remaining at 4°C for 30 min.

After the temperature sweep, the dynamic frequency sweep was carried out at 25°C using an angular frequency (ω) of 1 ~ 100 rad/s at a constant strain of 1%. The storage and loss modulus (G', G'') as functions of ω were recorded. Large‐scale deformation tests were performed at 25°C. The strain was increased from 1% up to the fracture point when the stress began to decrease at a frequency of 1 Hz. In addition, EMC gels were prepared in tubes under the same heating process. Each 3 ml aliquot of 30 mg/ml EMC was poured into a 5‐ml test tube and heated at 40, 60, 90, or 100°C for 10 min, followed by incubation overnight at 4°C to form gels.

In the case of starch‐containing myofibrillar gel, the minced eel muscle was homogenized with nine volumes (v/w) of chilled distilled water, filtered with gauze to remove the connective tissue, followed by centrifugation at 10,000 g for 20 min at 4°C. The myofibril was prepared according to the method described by Ref. (Bertram et al., 2004) with a slight modification. The EMC was extracted from the removed connective tissue with 0.1 mol/L NaOH as previously described. The myofibril and EMC had a final protein concentration of 50 and 30 mg/ml, respectively. The EMC was preheated at 80, 90, or 100°C for 10 min, and the samples were expressed as EMC‐80, EMC‐90, and EMC‐100, respectively. The myofibril (2.0 g), potato starch (0.8 g), and distilled water (1.0 g), with or without EMC (1.0 g), were mixed and homogenized well. The temperature sweep program was performed as follows: heating from 20 to 80°C at a rate of 5°C/min and incubation at 80°C for 10 min, followed by cooling from 80 to 4°C at the rate of 5°C/min and incubation at 4°C for 10 min.

### 2.11 Preparation of eel ball

The minced eel fillets (100 g), potato starch (40 g), salt (1 g), and EMC or collagenase solution (50 ml) were mixed and homogenized (600 g, 30 s, 6 times). The concentrations of EMC solution were 0, 0.01, and 0.02 g/ml. Twenty grams of the mixture was taken and rolled into a ball. The eel balls were heated at 90°C for 30 min followed by being cooled to room temperature and storage at 4°C overnight prior to analysis. As for the preparation of eel ball samples under different heat treatments, the heating temperatures were set to 60, 70, 80, 90, and 100°C.

### 2.12 Texture profile analysis (TPA) and gel strength

The texture and gel strength were measured by first cutting the eel balls into cylinders, each measuring 15 mm in height and 10 mm in diameter from the center (TA. XT Plus, Stable Micro System). A cylindrical stainless plunger (P/50) measuring 5 mm in diameter was used in the TPA tests. The following test conditions were selected for all measurements: a pretest speed of 5.0 mm/s, a test speed of 1.0 mm/s, and a post‐test speed of 1.0 mm/s, at a compression ratio of 50%. The data obtained from TPA curve were used to calculate the textural parameters. Among the TPA parameters, springiness was calculated as the ratio of the time from the start of the second area up to the second probe reversal divided by the time between the start of the first area and the first probe reversal. Cohesiveness indicates the difficulty in disrupting the internal structure of the samples. Chewiness was calculated as a product of hardness, cohesiveness, and springiness (Caner et al., 2008). Gel strength was measured using a cylindrical probe (P/5). The trigger force used was 5 g, with 1 mm/s of pretest and test speed. The cell load capacity of the texture analyzer was 30 kg, and the return distance was 40 mm. Gel strength was calculated as a product of breaking force (g) and deformation (mm) (Huda et al., 2011). Each measurement was replicated at least five times.

### 2.13 Masson staining of eel ball

Small pieces of eel ball were dissected, fixed in formalin solution for 24 hr, and embedded in paraffin. Five‐micrometer sections were cut with a microtome (JYD‐325; Yudian Medical Apparatus Factory). The prepared sections were stained with Masson stain (Fernandez et al., 1998). Subsequently, the tissue sections were dehydrated in alcohol, cleared in xylene, and mounted with a resin. The slides were visualized with a light microscope (DFC500; Leica). Muscle fibers and collagen fibers were stained red and blue, respectively.

### 2.14 Statistical analysis

Results of the analysis were expressed as means of at least three determinations ± standard deviation (*SD*). The differences in the results between different groups were determined with analysis of variance (ANOVA) and least significant difference (LSD) test using SPSS version 17.0 for Windows (SPSS Inc.). *p* < .05 was considered statistically significant.

## 3 RESULTS AND DISCUSSION

### 3.1 Heat‐induced structural changes of EMC

**FIGURE 1:**
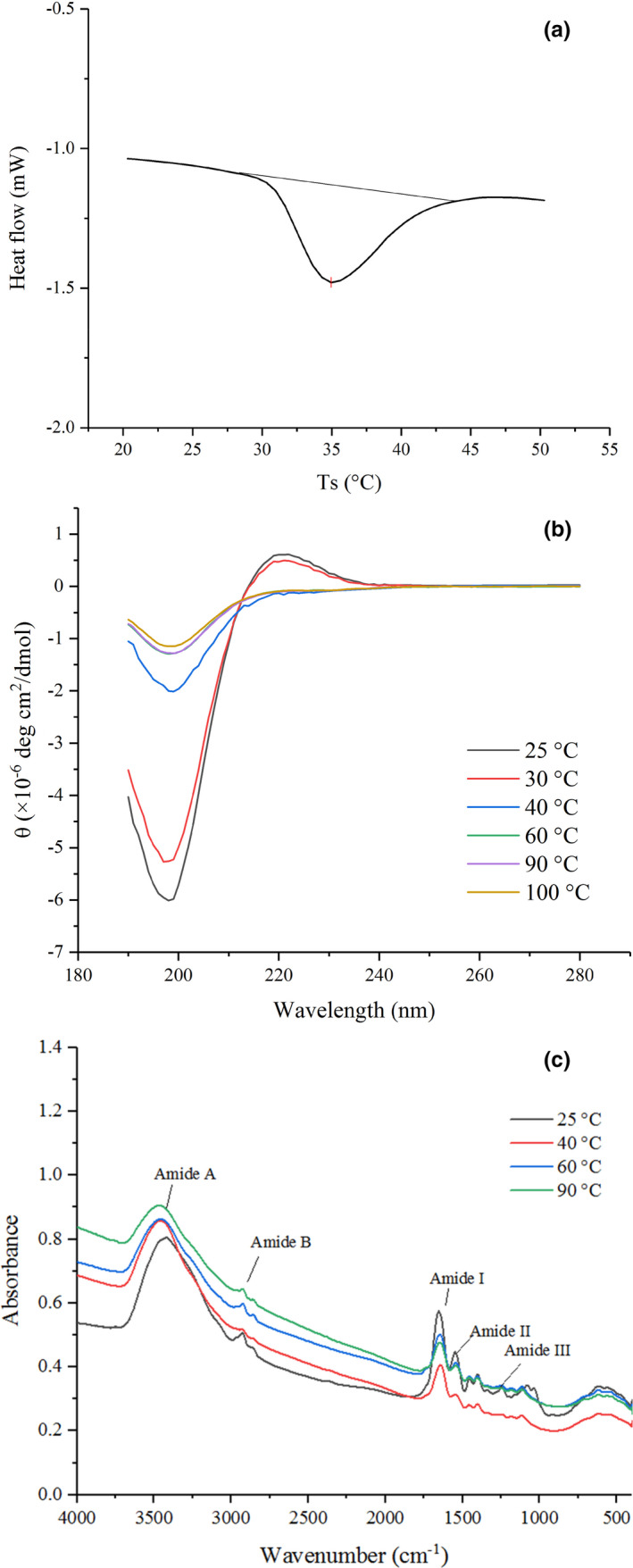
Heat‐induced structural changes of EMC. (a) DSC thermogram of EMC; (b) CD spectra of EMC; and (c) FTIR spectra of EMC

A DSC thermogram of EMC is presented in Figure 1a, from which it can be seen that the *T_d_* was 34.95°C and DH was −22.86 mJ. *T_d_* of EMC was higher than that of collagen from deep‐sea red‐fish bone (17.5°C) and puffer fish (28°C) but lower than that of collagen obtained from sea cucumber (57°C) and chicken sternal cartilage (43.8°C) (Cao & Xu, 2008; Nagai et al., 2002; Wang et al., 2008). The variation in *T_d_* of collagen between these species was attributed to their habitat, body temperature, and imino acid (proline and hydroxyproline) content. Collagen with higher imino acid usually has higher *T_d_* (Kittiphattanabawon et al., 2005).

The CD spectra of the EMC solution under heat treatment at 25–100°C and 190–280 nm is shown in Figure 1b. The EMC at 25°C had a positive peak at 222 nm and a negative peak at 198 nm, indicating the characteristic of triple helix conformation of collagen (Gopinath et al., 2014). The intensity of both peaks was decreased after heating at 30°C, suggesting progressive thermal unfolding and disassembly of collagen. As the temperature increased to 40°C and above, a significant decrease in magnitude and redshift of the negative peak was detected, along with a parallel loss of the positive peak. The CD spectra of EMC heated between 40 and 100°C did not significantly differ and were similar to previous reports (Lopes et al., 2015). These results indicated denatured EMC at temperatures above 40°C, with the loss of triple helix conformation. Moreover, the CD spectra suggested that the thermal denaturation temperature of EMC ranged between 30 and 40°C, which was consistent with DSC data (T_max_ 34.95°C).

To further investigate the structural changes of EMC under heating, the FTIR spectra of EMC heated at 25, 40, 60, and 90°C were measured (Figure 1c) and the wavenumber and assignment indication of peaks identified from FTIR spectra were investigated (Table 1). With the increase in heating temperature, the amide A shifted from a wave number of 3,414–3,464 cm^−1^ (blue shift), and the intensity was progressively weakened; the absorbance of the amide I also decreased progressively from 0.574 to 0.476. Because the absorbance of both amide A and amide I indicates the strength of hydrogen bonds between N‐H stretch and C=O (Pielesz, 2014), and the assignments and indications of peaks identified from FTIR spectra (Table 1) indicated the significant differences in the location of the peaks of amide A and amide I, these results of decreasing intensity of amide A and amide I demonstrated that progressive heating reduced the intra‐ and intermolecular interactions associated with hydrogen bonds in EMC.

**TABLE 1:** The assignment indication of peaks identified from Fourier transform infrared spectroscopy (FTIR) spectra

Region	Wavenumber(cm^−1^)				Assignment
	25°C	40°C	60°C	90°C	
Amide A	3,414 ± 1^c^	3,458 ± 3^b^	3,458 ± 1^b^	3,464 ± 2^a^	N‐H stretch couple with H‐bond, O‐H stretch
Amide B	2,928 ± 2^a^	2,929 ± 2^a^	2,926 ± 10^a^	2,928 ± 0^a^	CH_3_ asymmetrical vibration
Amide I	1,653 ± 1^a^	1,643 ± 0^b^	1,645 ± 2^b^	1,645 ± 0^b^	C=O stretch, COO‐stretch
Amide II	1,549 ± 2^a^	1,549 ± 0^a^	1,543 ± 3^b^	1,545 ± 1^b^	NH bend coupled with CN stretch
	1,454 ± 0^b^	1,458 ± 1^a^	1,456 ± 0^a^	1,458 ± 1^a^	CH_2_ deformation
	1,400 ± 1^b^	1,402 ± 0^b^	1,404 ± 1^a^	1,404 ± 0^a^	COO‐symmetrical stretch
	1,335 ± 3^b^	1,348 ± 1^a^	1,338 ± 4^b^	1,350 ± 2^a^	CH_2_ wag of proline and glycine; CO stretching in COO‐
Amide III	1,240 ± 2^b^	1,248 ± 3^a^	1,246 ± 2^a^	1,250 ± 1^a^	NH stretch coupled with CN stretch
	1,190 ± 0^a^	1,182 ± 2^b^	1,184 ± 2^b^	1,184 ± 2^b^	C‐O stretching
Fingerprint	1,078 ± 5^b^	1,117 ± 4^a^	1,115 ± 5^a^	1,117 ± 3^a^	C‐O stretch
	889 ± 6^a^	866 ± 5^c^	866 ± 3^c^	879 ± 3^b^	C‐H deformation of mannuronic acid
	613 ± 2^b^	619 ± 2^a^	619 ± 1^a^	619 ± 2^a^	Guluronic acid residue

Note

Within each row, means with different lowercase letters are significantly different (*p* < .05) among different groups.

The assignment of peaks was performed according to reports from Sow et al. (2019).

The CD and FTIR data together with the involvement of hydrogen bonds in helix (especially the collagen triple helix) and β‐sheet suggest that the heat treatment completely destroyed the triple helix conformation in EMC at 40°C, which was higher than T_max_ of EMC; heating at a temperature higher than 40°C further decreased the β‐sheet and α‐helix secondary structure due to the disruption of hydrogen bonds (Pielesz, 2014).

### 3.2 Physicochemical properties of heat‐treated EMC

#### 3.2.1 Heat treatment decreased surface hydrophobicity of EMC

**FIGURE 2:**
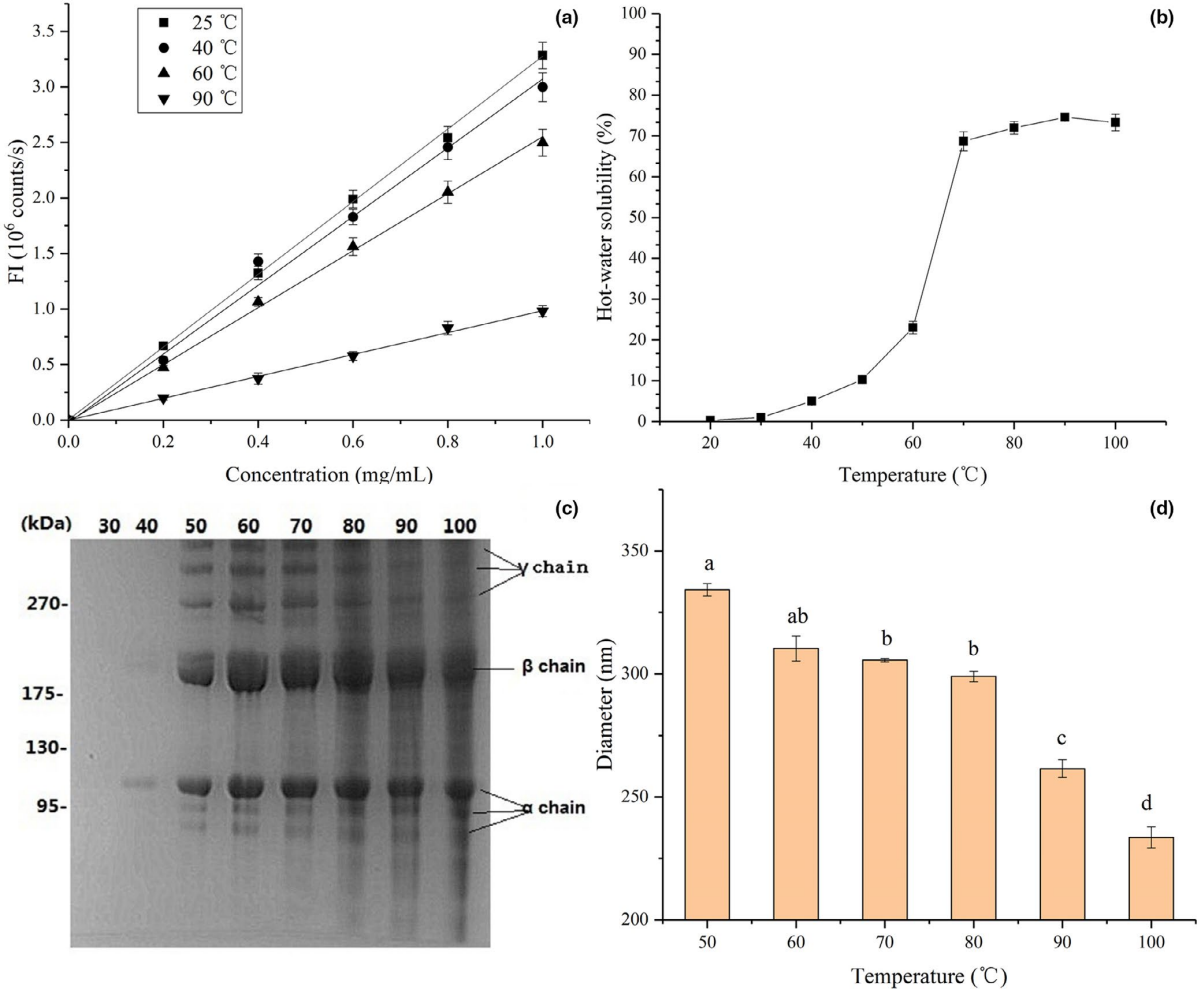
Physicochemical properties of EMC. (a) Protein surface hydrophobicity of EMC; (b) hot‐water solubility of EMC at different temperatures; (c) SDS‐PAGE electropherogram of the dissolved fraction; and (d) diameter distribution profiles of EMC treated at different temperatures. Columns with different lower case letters indicate significant differences, as assessed by the LSD test (*p* < .05)

The variation in protein surface hydrophobicity is highly correlated with changes in protein structure and function (Kato & Nakai, 1980). As shown in Figure 2a, untreated EMC at 25°C showed the maximum surface hydrophobicity with a hydrophobicity index of 3.27 ± 0.14. The surface hydrophobicity index of EMC after exposure to temperatures at 40 and 60°C decreased to 3.09 ± 0.09 and 2.56 ± 0.08, respectively, indicating the disintegration of EMC structure and the increased surface hydrophilicity. The EMC solution after heating at 90°C showed the minimum surface hydrophobicity index of 1.00 ± 0.10, which indicated further structural damage to EMC and external orientation of additional hydrophilic groups. The change in surface hydrophobicity of EMC at different temperatures was consistent with the results of the above mentioned changes in the EMC structure observed by CD and FTIR spectra (Figure 1b and c), which suggested the disruption of intra‐ and intermolecular hydrogen bonds. This phenomenon of collagen is totally different from almost axiomatic folding and assembly of globular protein, which is predominantly a hydrophobic effect. As shown in Figure 2a, heat treatment decreases surface hydrophobicity of EMC, which is different from the usual increment of surface hydrophobicity of globular protein after heat denaturation (Wang et al., 2017).

#### 3.2.2 Heat treatment increased hot‐water solubility of EMC, but caused collagen fibers to hydrolyze gradually

As shown in Figure 2b, the solubility of EMC was very low below 40°C but steadily and significantly increased from 70 to 100°C. The solubility of EMC was similar to that of the common carp *Cyprinus carpio* at 40–90°C, but was higher than that of kuruma prawn *Marsupenaeus japonicus* (Mizuta, Yoshinaka & Sato, 1995). The difference in hot‐water solubility of collagen was species‐dependent and related to their denaturation temperatures (Mizuta, Tanaka & Yokoyama, 1999). The hot‐water soluble fractions between 30 and 100°C were analyzed by SDS‐PAGE (Figure 2c). Three distinct α chains, together with their cross‐linking components including β and γ chains, were clearly detected in the SDS‐PAGE patterns, and the density of the protein bands of these collagenous components increased with hot‐water solubility. The SDS‐PAGE patterns of EMC were essentially similar at temperatures higher than 50°C, except for protein bands with molecular weights lower than that of β and α chains, which suggested heat‐induced degradation products of collagen, that is, gelatin. Overall, EMC samples with higher surface hydrophobicity displayed lower solubility, substantiated by the significant correlations between solubility and surface hydrophobicity (*r* = −.993). These results indicated that heat treatment of EMC decreases protein surface hydrophobicity and improves solubility and causes collagen to hydrolyze or gelatinize gradually as well.

#### 3.2.3 Heat treatment reduced the particle diameter of EMC

The average particle sizes of EMC with heat treatment from 50 to 100°C are shown in Figure 2d. At 50°C, the EMC exhibited the largest diameter (334 ± 12 nm), while treatment at 100°C yielded the smallest diameter (233 ± 9 nm). Collagen samples with a higher average particle size exhibited increased protein surface hydrophobicity, which was supported by the significant correlations between surface hydrophobicity and diameter (*r* = .995).

Combined with significant correlations between solubility and surface hydrophobicity (*r* = −.993), it is suggested that both average particle size and solubility of EMC vary with surface hydrophobicity, which is highly correlated with protein structure (Kato & Nakai, 1980). Therefore, the heat‐induced structural changes in EMC, including disassembly and degradation of triple helix, induce significant variation in physicochemical properties, such as surface hydrophobicity, solubility, and average particle diameter.

### 3.3 Rheological behavior of EMC gels

#### 3.3.1 Mild heating improved gelling of EMC, whereas overheating weakened the improvement

**FIGURE 3:**
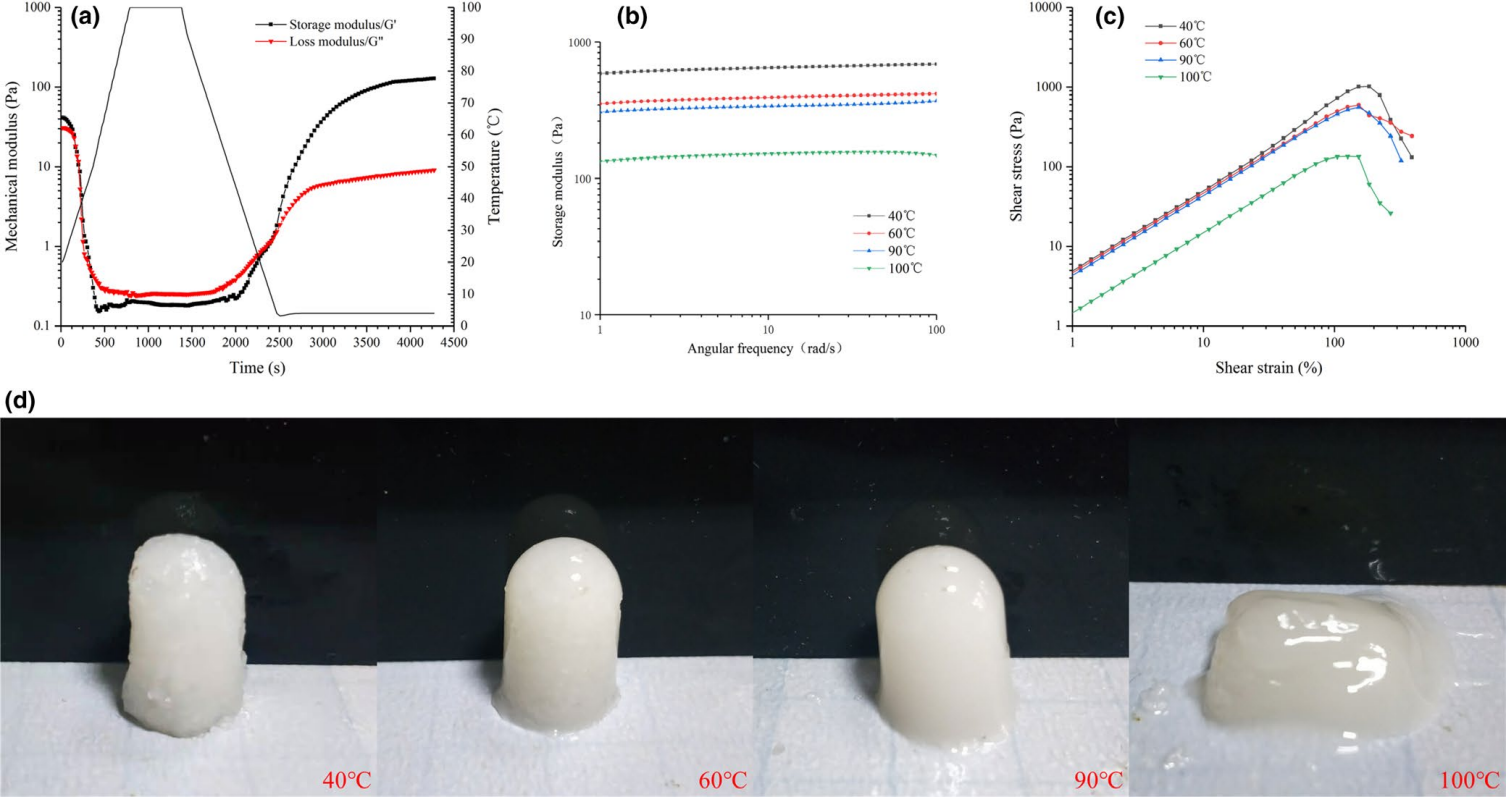
Effect of heating temperature on the rheological behavior (a–c) and performance (d) of EMC gels. (a) The mechanical moduli (G' and G") and temperature as a function of time in the gelling process of EMC heated at 100°C; (b) frequency sweep of the EMC gels heated at different temperatures; (c) the large deformation of the EMC gels heated at different temperatures. (d) Effect of heating temperature on the performance of EMC gels

Evolution of storage modulus (G'), loss modulus (G''), and temperature as a function of time in the gelling of EMC is shown in Figure 3a. The values of G' and G" at 40, 60, 90, and 100°C for 10 min showed a similar trend (for brevity, only treatment at 100°C is shown in the figure). G' and G" decreased significantly in the heating‐up phase and changed slowly at the heat conservation stage, followed by a significant increase at the cooling stage and eventual stabilization at the cold storage stage suggesting that the EMC transformed gradually from solution to the solid gel structure during the cooling. The gel network structure was enhanced during the cold storage probably due to the hydrogen bonds favored at low temperatures, driving collagen assembly (Leikin et al., 1995; Wang et al., 2017). Loss tangent (Tan δ) refers to the ratio of G" to G'. The gel formation temperature (T_gel_) is defined as the temperature point when Tan δ equals 1(Wang et al., 2019). As shown in Table 2, the T_gel_ values of EMC heated at 40, 60, 90, or 100°C for 10 min were 21.21, 24.98, 12.53, and 5.27°C, respectively. As the heat treatment exceeds 60°C, the gel formation temperature is decreased, which might be correlated with the disintegration of fiber structure and molecular degradation of EMC at high temperature. The lower T_gel_ value of EMC at 40°C compared with 60°C is facilitated by the incomplete dissolution of EMC at 40°C, according to the data presented in Figure 2. In addition, the final G' values of the gel under the four treatment conditions were 592.66, 386.97, 295.28, and 113.31 Pa. The lowest T_gel_ value and final G' of EMC treated at 100°C suggest obvious heat‐induced degradation at 100°C (Figure 2c), resulting in a weaker gel network.

**TABLE 2:** Effect of heating temperature on EMC gel properties

Parameters	40°C	60°C	90°C	100°C
Final G' (Pa)	592.66 ± 20.49^a^	386.97 ± 17.63^b^	295.28 ± 8.47^b^	113.31 ± 7.58^c^
Final G" (Pa)	17.49 ± 3.25^a^	13.72 ± 4.45^a^	9.13 ± 1.25^b^	6.42 ± 2.16^c^
T _gel_ (°C)	21.21 ± 3.02^a^	24.98 ± 2.74^b^	12.53 ± 1.34^c^	5.27 ± 0.76^d^
Breaking force (Pa)	1,013.66 ± 48.39^a^	594.16 ± 27.56^b^	557.90 ± 32.43^b^	134.51 ± 18.89^c^
Corresponding strains (%)	184.3 ± 5.3^a^	153.0 ± 3.7^b^	153.3 ± 2.8^b^	126.5 ± 4.2^c^

Note

Values represent means of three experiments, and the different lower case letters (a–d) indicate significant differences in the same row as assessed by an LSD test (*p* < .05).

In order to further elucidate the differences in viscoelasticity and force of EMC gels treated at different temperatures, a frequency scanning experiment (small deformation) was carried out on the gels, and the results are shown in Figure 3b. All samples showed a weak frequency dependence. Based on the theory of polymer dynamics governing liquid‐like fluid, the frequency dependence of G' value shows a power relation. Therefore, the power law model G' = aω^b^ can be used to fit the relationship between G' and ω (oscillation frequency). The b value represents the properties of the force acting on the gel structure formation. The b values of EMC gel treated at 40, 60, 90, and 100°C are 0.0323, 0.0346, 0.0342, and 0.0436, respectively, indicating that they were physical gels exhibiting the characteristics of elastic gels composed of hydrogen bonds (Hesarinejad et al., 2004).

Large deformations of EMC gels pretreated by different temperatures are shown in Figure 3c. For EMC gel samples pretreated at 40, 60, 90, and 100°C, the breaking forces were 1,013.66, 594.16, 557.9, and 134.51 Pa, and the corresponding strains were 184%, 153%, 153%, and 126%, respectively (Table 2). In the test, the yield stress reflects the hardness of the gel, and the yield strain indicates the deformability or brittleness (Weijers et al., 2006). The large deformation sweep indicated that the heat treatment at 100°C significantly decreased the hardness and deformability of EMC gel compared with those treated at 40, 60, and 90°C. The high‐intensity heat treatment at 100°C resulted in structural destruction of EMC, including disassembly of triple helix and degradation, as shown by FTIR data, CD (Figure 1b and c), and SDS‐PAGE results (Figure 2c).

The performance of EMC gels under different heat treatments is shown in Figure 3d. It was found that after heat treatment at 40°C, the EMC gel had a rough appearance, with visible coarse fibers inside, owing to incomplete dissolution of EMC based on the results of hot‐water solubility (Figure 2). The gels exhibited smooth and translucent surface when heated at 60 and 90°C. However, at 100°C, EMC failed to form a shaped gel, indicating a decrease in gel hardness, which was consistent with rheological analysis (Figure 3).

### 3.4 Effects of EMC on the texture of a mimic of eel ball

**FIGURE 4:**
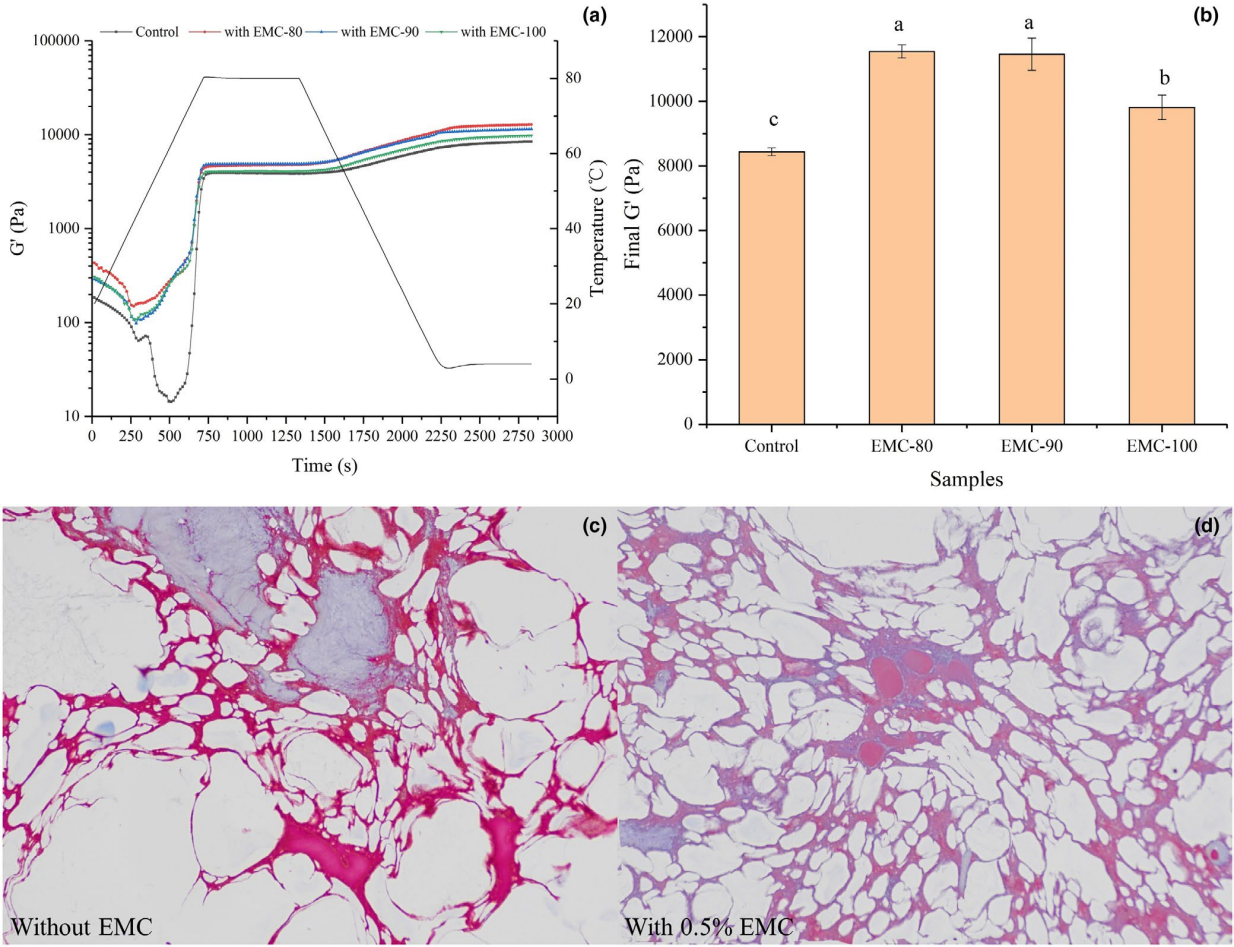
Effects of EMC on the dynamic rheological properties of a mimic of eel ball and the microstructure of eel ball. Storage modulus (G') (a) and final G' (b) of the starch‐containing myofibril gel supplemented with EMCs preheated at different temperatures. Columns with different lower case letters indicate significant differences in LSD test results (*p* < .05); Masson image (×400) of eel ball without (c) or with (d) the addition of 0.5% EMC

To further investigate the effects of EMC on the texture of eel ball, the parameter G' was used to investigate the effects of EMC pretreated at different temperatures on the dynamic rheological properties of the starch‐containing myofibrillar gel, a mimic of eel ball. Figure 4a shows the G' curves of the control group and the three groups treated with EMC at 80, 90, and 100°C. During the heating phase, G' of all samples gradually decreased and then increased significantly.

As shown in Figure 4b, the final G' of the starch‐containing myofibrillar gels with EMC was significantly higher than that of the control group, indicating that EMC enhanced the gel properties of the starch‐containing myofibrillar gel. This result may be attributed to the excellent solubility and gelling ability of EMC after heating and its association with the starch‐containing myofibrillar gel, as well as interaction with starch (Inaba et al., 1995). However, the final G' of the EMC‐100 sample was significantly (*p* < .05) lower than that of the EMC‐80 and EMC‐90 samples, suggesting that the treatment at 100°C attenuated the effect of EMC on starch‐containing myofibrillar gel. The obvious thermal degradation of EMC at 100°C significantly decreased the gelling ability of EMC, as shown in Figure 3d. These results indicated EMC improved the dynamic rheological properties of starch‐containing myofibrillar gels with EMC, a mimic of eel ball, but the treatment of EMC at 100°C attenuated the effect.

### 3.5 Effect of EMC on texture improvement of eel ball

The effects of EMC and collagenase addition on the texture of eel ball were investigated by TPA analysis. As shown in Table 3, the texture of eel ball was improved by the addition of EMC. Gel strength, springiness, and chewiness of eel ball containing 0.25% or 0.50% EMC were significantly higher than those of the control (*p* < .05), and the enhancing effects demonstrate EMC content dependence. Similar results were observed in sardine surimi containing duck feet collagen (Huda et al., 2011). Du and Betti (2016) reported that hydrophilic groups in collagen molecules might stabilize the water surrounding the myofibrils, thereby contributing to the improvement of myofibrillar protein stability. In addition, the addition of collagen to potato starch effectively increased the hardness and slightly increased the cohesiveness of the starch gel (Inaba et al., 1995). This suggests that interactions with myofibrils and starch may contribute to the effect of EMC supplementation on the texture of the eel ball. To confirm the improved texture of eel ball induced by EMC, collagenase was added to degrade EMC. As a result, the gel strength, springiness, cohesiveness, and chewiness of the eel ball were significantly decreased (*p* < .05), suggesting that the hydrolysis of EMC during processing led to the softening of eel ball. These results are consistent with the impact of EMC on the dynamic rheological properties of starch‐containing myofibrillar gel (Figure 4a and b).

**TABLE 3:** Results of texture analysis of eel ball supplemented with collagen or collagenase

Samples	Gel strength (g)	Springiness	Cohesiveness	Chewiness
Eel ball control	411.19 ± 1.00^c^	0.79 ± 0.01^b^	0.56 ± 0.01^b^	540.20 ± 14.72^b^
With 0.25% EMC	440.06 ± 0.87^b^	0.81 ± 0.00^a^	0.64 ± 0.02^a^	574.83 ± 11.54^b^
With 0.50% EMC	485.66 ± 4.13^a^	0.83 ± 0.00^a^	0.63 ± 0.02^a^	772.20 ± 41.65^a^
With Collagenase	382.00 ± 2.87^d^	0.74 ± 0.01^c^	0.48 ± 0.03^c^	301.91 ± 20.58^c^

Note

EMC‐Eel muscle collagen. Values represent means of three experiments, and the different lower case letters (a–d) indicate significant differences in the same row based on LSD test (*p* < .05).

#### 3.5.1 Supplementation of EMC significantly improved uniformity and tightness of the microstructure of eel ball

The microstructures of eel ball with and without the addition of EMC are presented in Figure 4c and d. Masson staining is a common method used to distinguish myofibril from collagen. As shown in Figure 4 c and d, myofibril (red) and collagen (blue) together constituted the network structure of eel ball, with starch granules filled within the network (white parts). The eel ball with EMC exhibited a more uniform and tighter network than the control, which carried large holes. A more uniform network structure usually indicates a better texture of the eel ball, as fish balls with large holes are more likely to deform and dehydrate (Tee & Siow, 2014). The results of Masson staining further illustrate the important role of EMC in the texture of the eel ball. It is speculated that during the heating, the structural changes of EMC enhance its solubility and hydrophilicity, to facilitate dispersion in the mixture and interaction with starch and myofibrils, resulting in improved gel network of the eel ball. In addition, the gelling ability of EMC is also conducive to texture development of the eel ball.

### 3.6 Schematic illustration

Myofibril in fish muscle has sulfhydryl groups in the myosin globular head, which makes a large contribution to the aggregation of myosin and texture properties of myofibrillar gel (Zhao et al., 2019; Zhou & Yang, 2019). However, collagen is devoid of sulfhydryl group and myosin head, which implies that disulfide bond formation is not critical for collagen gelation (Sun & Holley, 2011). The data in Figure 3b indicated that EMC acted as a physical gel, supporting that disulfide bond was not the main force in gelation. Moreover, collagen lacks interaction with fish myofibrillar protein (Feng, Fu, et al., 2017). Therefore, it was hypothesized that EMC mostly contributed via self‐gelation, interaction with starch, and filler effect to the texture development of eel ball, based on the results in Figure 3 and Figure 4a, d.

The data of DSC, CD, and FTIR (Figure 1; Table 1) confirmed the heat‐induced structural changes in EMC. That was when the heating temperature exceeds *T_d_*, the hydrogen bonds within and between EMC molecules decreased, and EMC underwent denaturation with a significant decrease of α‐helix and β‐sheet, which in turn even leaded to the degradation of polypeptide chains of EMC (Figure 2c).

**FIGURE 5:**
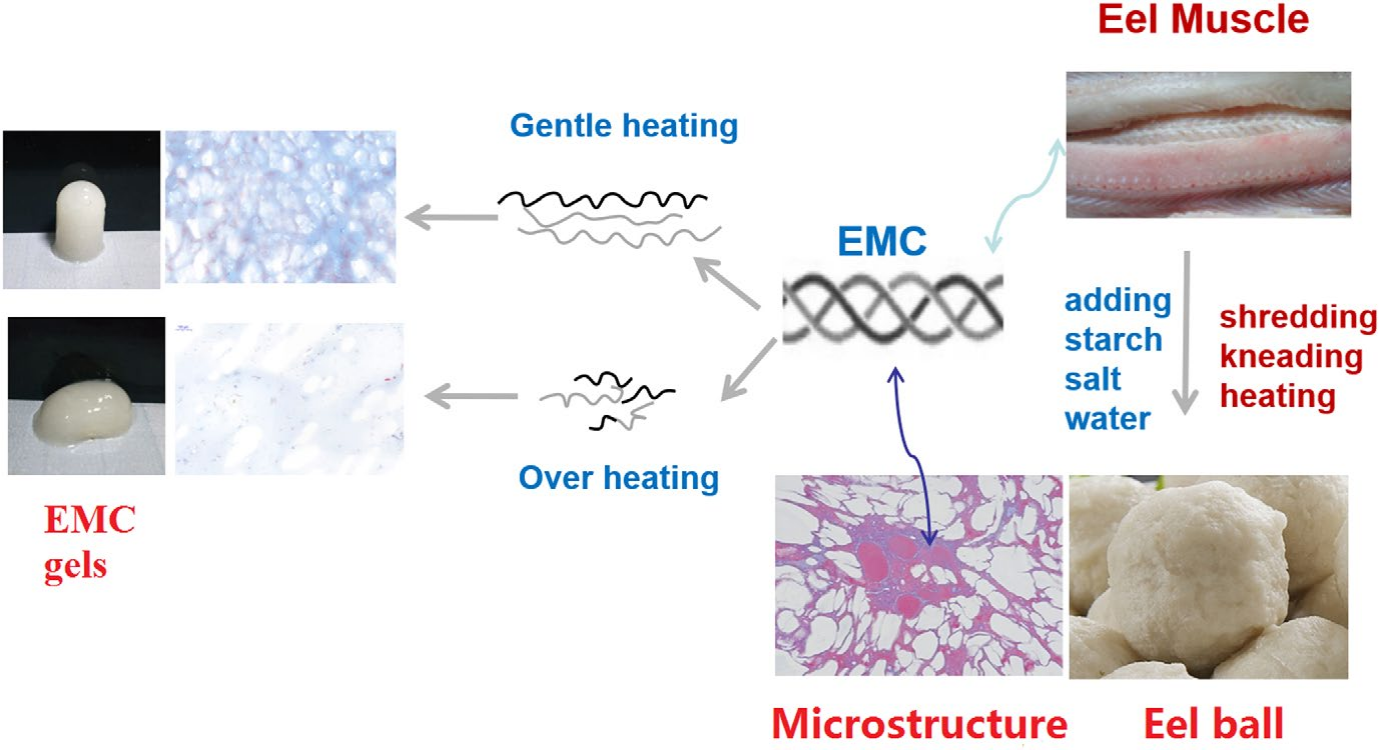
Schematic illustration of the relationship of heat‐induced structural changes in eel muscle collagen (EMC) with texture development in eel balls. In the microstructure of eel ball, myofibril (red) and collagen (blue) together constituted the network structure of eel ball, with starch granules filled within the network (white parts). EMC, with gentle heat treatment, can improve the microstructure of eel balls via gelling ability, filler effect, and interaction with starch; however, overheating may weaken this improvement because overheat‐induced degradation of EMC decreases its gelling ability significantly

As a result of these structural changes, two aspects of the physicochemical properties of EMC vary significantly: (1) Hydrophilicity and solubility increase (Figure 2a, b) and the particle size becomes smaller (Figure 2d), which may contribute to the filler effect and self‐gelation and enhance the gel strength and texture (Table 2); and (2) the degradation of EMC caused by heat treatment reduces the molecular length of EMC and weakens its gelling ability (Figure 3d), gel strength, and texture (Tables 2 and 3) (Sun & Holley, 2011). Based on the abovementioned hypothesis, these two aspects of physicochemical properties of EMC have to compromise, that is, the heat treatment that causes structural changes must be moderate, to ensure the optimal texture development of the eel balls, as shown by the schematic illustration in Figure 5. The optimal heat treatment at 90°C for 30 min found in our works is an evidence supporting such hypothesis.

## 4 CONCLUSIONS

The application of fish collagen or gelatin as an additive has been investigated previously with gel weakening effect on the texture of most myofibrillar gel (Sun & Holley, 2011), but our results indicated the strengthening effect of intrinsic collagen on gel texture of eel ball and that mild heating of EMC improved the textural properties of eel ball, resulting from heat‐induced structural and physiochemical changes of EMC to enhance the network structure of eel balls. However, overheat‐induced degradation of EMC weakened the strengthening effect. Therefore, the texture development of eel ball can be regulated by heat‐induced structural changes, as well as structure–function relationship of collagen, compared with previous studies on myofibrillar proteins and exogenous gelatin (Feng, Zhu et al., 2017; Zhang et al., 2013). Furthermore, because eel ball is a typical representative of fish balls, and fish balls are a type of heat‐induced gel of fish surimi supplemented with starch and salt, this study may provide texture‐related insights to manage the processing and quality control of fish balls and diverse heat‐treated products of fish surimi containing collagen.

## CONFLICT OF INTEREST

The authors have no conflict of interest to declare.

## AUTHOR CONTRIBUTION

**Yafei Wang:** Data curation (equal); Formal analysis (equal); Investigation (lead); Methodology (supporting); Resources (supporting); Software (supporting); Validation (equal); Writing‐original draft (lead). **Xufeng Wang:** Data curation (supporting); Formal analysis (equal); Investigation (supporting); Methodology (equal); Resources (supporting); Software (equal); Validation (equal); Writing‐review & editing (supporting). **Chuntong Lin:** Formal analysis (equal); Methodology (equal); Resources (equal); Software (equal). **Mengqin Yu:** Formal analysis (supporting); Investigation (supporting); Validation (supporting). **Shanshan Chen:** Formal analysis (supporting); Investigation (supporting); Resources (supporting). **Jingke Guo:** Formal analysis (supporting); Methodology (supporting); Software (supporting); Writing‐review & editing (supporting). **Pingfan Rao:** Conceptualization (supporting); Writing‐review & editing (supporting). **Song Miao:** Resources (supporting); Writing‐review & editing (supporting). **Shutao Liu:** Conceptualization (lead); Data curation (equal); Formal analysis (equal); Funding acquisition (lead); Investigation (equal); Methodology (equal); Project administration (lead); Resources (lead); Software (supporting); Supervision (lead); Validation (equal); Writing‐original draft (equal); Writing‐review & editing (lead).

## Data Availability

Research data are not shared.
